# Identifying and evaluating a disulfidptosis-related gene signature to predict prognosis in colorectal adenocarcinoma patients

**DOI:** 10.3389/fimmu.2024.1344637

**Published:** 2024-06-19

**Authors:** Ming Li, Jin Wang, Yuhao Zhao, Changjie Lin, Jianqing Miao, Xiaoming Ma, Zhenyu Ye, Chao Chen, Ke Tao, Pengcheng Zhu, Qi Hu, Jinbing Sun, Jianfeng Gu, Shaohua Wei

**Affiliations:** ^1^ Department of General Surgery, The Second Affiliated Hospital of Soochow University, Suzhou, China; ^2^ Department of General Surgery, Changshu Hospital Affiliated to Soochow University, The First People’s Hospital of Changshu, Changshu, Jiangsu, China; ^3^ School of Public Health, Suzhou Medical College of Soochow University, Suzhou, Jiangsu, China; ^4^ Department of Biliary and Pancreatic Surgery, Renji Hospital Affiliated to Shanghai Jiaotong University School of Medicine, Shanghai, China; ^5^ Department of Breast Surgery, Fudan University Shanghai Cancer Center, Shanghai, China

**Keywords:** disulfidptosis, LASSO, prognostic model, cell senescence, POU4F1

## Abstract

Disulfidptosis, a regulated form of cell death, has been recently reported in cancers characterized by high SLC7A11 expression, including invasive breast carcinoma, lung adenocarcinoma, and hepatocellular carcinoma. However, its role in colon adenocarcinoma (COAD) has been infrequently discussed. In this study, we developed and validated a prognostic model based on 20 disulfidptosis-related genes (DRGs) using LASSO and Cox regression analyses. The robustness and practicality of this model were assessed via a nomogram. Subsequent correlation and enrichment analysis revealed a relationship between the risk score, several critical cancer-related biological processes, immune cell infiltration, and the expression of oncogenes and cell senescence-related genes. POU4F1, a significant component of our model, might function as an oncogene due to its upregulation in COAD tumors and its positive correlation with oncogene expression. *In vitro* assays demonstrated that POU4F1 knockdown noticeably decreased cell proliferation and migration but increased cell senescence in COAD cells. We further investigated the regulatory role of the DRG in disulfidptosis by culturing cells in a glucose-deprived medium. In summary, our research revealed and confirmed a DRG-based risk prediction model for COAD patients and verified the role of POU4F1 in promoting cell proliferation, migration, and disulfidptosis.

## Introduction

Colon adenocarcinoma (COAD) is the third most common cancer and has the second highest cancer-related mortality rate worldwide. Compared with USA and Japan, China has the highest all-age incidence for both sexes combined (1). Early-stage COAD has a 5-year survival rate of more than 90% after treatment; however, even after comprehensive treatment with surgery, radiotherapy, chemotherapy, molecular targeted therapy, and immunotherapy, for late-stage patients with distant metastases, the rate is still only 14% (2–4). In the clinical treatment of COAD, the TNM staging system has been the most commonly applied method for predicting patient prognosis in recent decades, but this method has limitations. Therefore, it is imperative to develop a molecular predictive system to help clinicians determine treatment options and drug choices for COAD patients.

Disulfidptosis is a form of regulated cell death (RCD) reported in cancers characterized by a high expression of solute carrier family 7 member 11 (SLC7A11) (5). Disulfidptosis mainly originates from the process by which nicotinamide adenine dinucleotide phosphate (NADPH) fails to reduce cystine to cysteine, which induces disulfide stress and actin cytoskeleton protein disulfide bond cross-linking and cytoskeleton contraction and ultimately induces disulfidptosis (6, 7). Disulfidptosis is triggered when cancer cells with high SLC7A11 expression are subjected to glucose starvation, and disulfidptosis-related genes (DRGs) were identified via CRISPR–Cas9 screening (8, 9). In preclinical models, treatment with a glucose transporter (GLUT) inhibitor can effectively inhibit glucose uptake, induce disulfidptosis in SLC7A11*^high^*-expressing cancer cells, and limit the growth of SLC7A11*^high^* cancer cells, such as UMRC6 kidney cell carcinoma xenografts in mice, which highlights the need for the development of cancer treatment strategies (8, 9). The interactions of tumor-related genes (TRGs) in the tumor microenvironment (TME) affect the survival, growth, migration, and adhesion of cancer cells. This study is based on the hypothesis that disulfidptosis, a form of cell death associated with high SLC7A11 expression, plays a significant role in colorectal adenocarcinoma (COAD) and can be used to predict prognosis through a specific gene signature based on DRGs.

Based on technological developments in transcriptomics and bioinformatics, such as CRISPR–Cas9 screening, bulk RNA-seq, and scRNA-seq, prognostic models of malignant tumors have been established to help determine the prognosis of cancer patients, but, to date, few DRG prognostic models of COAD have been reported. In our research, we constructed a molecular prognostic model for COAD based on DRGs by least absolute contact and selection operator (LASSO) and Cox regression analysis with transcriptomic and clinical data from COAD patients in the TCGA, GEO, and DRG databases. After accuracy and specificity validation, we constructed a novel disulfidptosis-related prognostic model that could predict the prognosis of COAD patients via the DRG-related risk score, which can be explained by the analyses of biological effects such as immune infiltration, specific tumorigenic pathways, and drug response and synergy. The aim of this study was to establish a solid platform for devising patient-specific treatment regimens and assisting clinicians in the prognostic assessment and clinical treatment of COAD patients. Additionally, the key gene POU4F1 in the model was further validated by *in vitro* assays.

## Materials and methods

### RNA-sequencing data and bioinformatics analysis data collection

The data and clinical information of 454 CRC patients and 92 normal colon tissue samples were obtained from the TCGA database (https://portal.gdc.cancer.gov). The data of 585 CRC patients and 19 nontumoral patients (GSE39582) were downloaded from the GEO database (https://www.ncbi.nlm.nih.gov/geo/). DRGs were extracted based on CRISPR–Cas9 screens from Gan’s study (5). Genes, including 32 synergists and 63 suppressors, were identified according to the criteria of a |NormZ value| >2 and a *P*-value <0.05 for further construction of the prognostic model.

### Construction and validation of a prognostic model based on DRGs

After the GSE39582 data were integrated, we used the “care” package to randomly subdivide the patients into two datasets at a ratio of 7:3 according to their survival status; these datasets were used as training sets and internal test sets, respectively. A total of 555 patients in the TCGA database were used as independent validation sets. DRGs downloaded and identified from CRISPR–Cas9 screenings were obtained from a previous study (5, 6). Gene expression data from the patients were used to identify DEGs, and least absolute shrinkage and selection operator (LASSO) regression analysis and multivariate Cox regression were used to construct the prognostic model. The risk score for each COAD patient was calculated based on the expression of DRGs (Exp_i_) and Cox coefficients (coef_i_), $Riskscore={\sum_{i=1}^{n}Exp}_{i}\times coef_{i}$ . We used the “glmnet” package for LASSO regression model analysis. Patients with COAD were divided into a high-risk group and a low-risk group according to the median risk score. We used the “survival” and “survminer” packages to perform univariate and multivariate Cox analyses, generate Kaplan−Meier plots, and estimate whether the risk score was an independent factor of clinicopathological features. To assess the prognosis of both groups, OS was analyzed via Kaplan−Meier curves. The prognostic ability of the risk model was evaluated by time-dependent receiver operating characteristic (ROC) curve analysis using the “survival ROC” software package. We investigated the ability of the prognostic model to predict the outcome of CRC patients by using the “TimeROC” package to generate a time-dependent receiver operating characteristic (ROC) curve. The area under the curve (AUC) of the ROC curve was calculated with the “survivalROC” package. Nomogram plots were generated with the “rms” package. To verify the DRG signature, the risk score of COAD patients in the TCGA dataset was used to verify the accuracy of the model. The risk score of COAD patients in the GSE39582 dataset was determined via the same method to verify the accuracy of the model.

#### Establishment of a prognostic nomogram for COAD

In the training set and test set, the associations between the DRG signatures and clinicopathological features were analyzed with the “rms” package. In addition, both univariate and multivariate Cox regression analyses were conducted to explore whether the risk score has an independent prognostic value in patients with COAD. The probabilities of 1-, 2-, 3-, 4-, and 5-year OS in COAD patients were assessed by clinical variables and risk scores. The accuracy of the nomogram was evaluated by the concordance index (CI) and calibration curve.

### Determination of DRGs’ differential expression

The differentially expressed genes (DEGs) were identified by the “limma” package with a |threshold of log (fold change)| >1 and a *P*-value <0.05 between the low and high groups.

### Enrichment analysis

Based on the correlation analysis between the risk score and all mRNAs, gene set enrichment analysis (GSEA) was further performed by using the “ClusterProfiler” package of R software (version 4.3.1).

In addition, the differentially expressed genes (DEGs) between the low and high groups were identified based on the R package “limma” with the thresholds of log(fold change) >1 and *P*-value <0.05. The DEGs were further input into the DAVID online tool (https://david.ncifcrf.gov/) for pathway and biological process enrichment.

### Correlation analysis

To further explore the biological function and clinical relevance of the DRG prognostic model, we performed a correlation analysis to evaluate the associations between the risk score and the expression of oncogenes, tumor mutation burden (TMB), immune regulatory gene expression, immune cell infiltration, and tumor immune dysfunction and exclusion (TIDE) score. This analysis utilized the Spearman method with the “psych” package.

Oncogene data were sourced from the ONGene database (http://www.ongene.bioinfo-minzhao.org) (10), while 73 immunomodulatory genes (IMGs) (11) were derived from earlier research. The immune cell infiltration score was calculated with the XCELL algorithm (12). Furthermore, the TIDE score, dysfunction score, and exclusion score for each dataset patient were estimated using the standard process with the TIDE online tool (http://tide.dfci.harvard.edu/) (13).

The Sanger Research Institute created the Genomics of Drug Sensitivity in Cancer database (GDSC) to gather information on tumor cell sensitivity and response to drugs (14). “OncoPredict” was employed to determine the drug sensitivity of each sample in the training and validation datasets, leveraging the GDSC V2.0 database (15).

### Cell lines and culture

The human colon adenocarcinoma cell line SW480 (SW-480), which was isolated from the large intestine of a Dukes C colorectal cancer patient, was obtained from the National Collection of Authenticated Cell Culture at the Chinese Academy of Science (Shanghai, China). Colon adenocarcinoma HCT116 cells (ab255451) were isolated from the colon of an adult male with colon adenocarcinoma obtained from the Abcam Trading (Shanghai, China). The SW480 cells were cultured in DMEM supplemented with 10% fetal bovine serum (FBS) and 1% penicillin−streptomycin from Thermo Fisher Scientific (Shanghai, China). The HCT116 cells were cultured in McCoy’s 5A medium supplemented with 10% FBS (Gibco) and 1% penicillin−streptomycin (Gibco). All cells were incubated at 37°C with 5% CO_2_ for culture and passage unless otherwise stated. For the glucose deprivation experiments, cells were cultured in glucose-free DMEM supplemented with dialyzed FBS as previously described.

### Short hairpin RNA construction, plasmid vectors, and transfection

The POU4F1 sequences of the primers used were as follows:

Forward: 5′ - ACGCACGAACTGAGTCGAAA - 3′
Reverse: 5′-CACTTCCCGGGATTGGAGAG-3′

The POU4F1 shRNA plasmid (sc-29839-SH) was purchased from Santa Cruz Biotechnology. The transfection of plasmid vectors was carried out in Opti-MEM (Invitrogen) using Lipofectamine 3000 reagent (Invitrogen) according to the manufacturer’s transfection protocol.

### Transwell migration assays and Transwell invasion assays

For the Transwell migration assay, cells were seeded in the upper chamber of a Transwell membrane (Corning, Inc., USA) with 200 µL of FBS-free medium, and 600 µL of complete medium was added to the lower chamber. After the cells were cultured at 37°C for 24 h, they were fixed with 4% paraformaldehyde and stained with 0.5% crystal violet solution. Subsequently, the cells in the upper chamber of the Transwell membrane were removed. Images of the migrated cells were captured under an inverted microscope and were then assessed using NIH ImageJ software (version 1.8.0).

### Western blotting and antibodies

Total protein was extracted from cells by using RIPA lysis buffer (Beyotime, China) and quantitated by using Enhanced BCA Kit (Beyotime, China). Total protein (30 µg) was separated via SDS−PAGE and transferred onto PVDF Transfer Membranes (Thermo Fisher Scientific, China). After blocking with 5% BSA, the membrane was incubated at 4°C overnight with primary antibodies against POU4F1 (PA5–41509) and beta-actin (MA5–15452), which were purchased from Thermo Fisher Scientific (Shanghai, China). Following the primary incubation, the membranes were incubated with HRP-labeled secondary antibodies. The protein bands were visualized using enhanced chemiluminescence (ECL) substrate and the GeneTools GBox (Syngene) system, the intensity of each band was quantified using ImageJ software (National Institutes of Health), and beta-actin was used as the internal control.

### β-gal fluorescence imaging

The cell aging detection reagent SPiDER-β-gal was used for β-gal fluorescence staining. Briefly, after the cells were washed with wash buffer, SPiDER-β-gal staining solution was added. The plate was incubated in the dark for 15 min, and the cells were washed twice with PBS, followed by observation and imaging under a fluorescence microscope.

### Disulfidptosis assay

Glucose-free DMEM was used to simulate glucose deprivation conditions. When POU4F1 was knocked down or overexpressed in cells, the culture medium was replaced with a glucose-free medium, and the regulatory effect of the gene on disulfidptosis was determined by measuring cell viability and apoptosis.

### Statistical analysis

All statistical analyses were performed using R software (version 4.1.3). Continuous variables were tested by Student’s *t*-test, while categorical variables were tested by chi-square test. A *p*-value <0.05 was considered significant.

## Results

### Data collection

Three COAD cohorts and corresponding clinical data were obtained from the TCGA and GEO databases (GSE39582). The demographic and clinical data for the training, internal testing, and independent validation sets are summarized in **Table 1**. After ruling out the samples with missing clinical information in the GEO (584 patients) dataset, the samples were randomly divided into a training set (*n* = 393, 70%) and an internal testing set (*n* = 168, 30%). As expected, no significant differences were found in the major clinicopathological features between the training, testing, and entire GEO datasets (*p* > 0.05) (**Table 1**).

**Table 1:** Clinicopathological features of the GSE39682 and TCGA_COAD datasets.

Characteristics		GSE39582				TCGA_COAD
		Training	Testing	All	*P*-value	
Age	≤60	107 (27.23%)	50 (29.76%)	160 (27.40%)	0.808	160 (28.83%)
	>60	286 (72.77%)	118 (70.24%)	424 (72.60%)		395 (71.17%)
Gender	Female	179 (45.43%)	74 (44.05%)	263 (44.96%)	0.955	264 (47.57%)
	Male	215 (54.57%)	94 (55.95%)	322 (55.04%)		291 (52.43%)
Location	Proximal	153 (38.83%)	67 (39.88%)	232 (39.79%)	0.949	NA
	Distal	241 (61.17%)	101 (60.12%)	351 (60.21%)		
MMR	pMMR	306 (85.00%)	138 (88.46%)	459 (85.63%)	0.574	NA
	dMMR	54 (15.00%)	18 (11.54%)	77 (14.37%)		
TP53_MUT	WT	105 (43.21%)	54 (50.94%)	161 (45.87%)	0.410	NA
	MU	138 (56.79%)	52 (49.06%)	190 (54.13%)		
KRAS_MUT	WT	224 (58.79%)	101 (63.12%)	328 (60.18%)	0.643	NA
	MU	157 (41.21%)	59 (36.88%)	217 (39.82%)		
BRAF_MUT	WT	321 (89.92%)	138 (91.39%)	461 (90.04%)	0.865	NA
	MU	36 (10.08%)	13 (8.61%)	51 (9.96%)		
CIN_status	Negative	72 (22.22%)	36 (26.47%)	112 (23.28%)	0.616	NA
	Positive	252 (77.78%)	100 (73.53%)	369 (76.72%)		
TNM_stage	Stage I/II	202 (51.27%)	92 (54.76%)	309 (52.82%)	0.739	307 (55.12%)
	Stage III/IV	192 (48.73%)	76 (45.24%)	276 (47.18%)		250 (44.88%)
TNM_M	M0	338 (88.95%)	141 (88.12%)	499 (89.11%)	0.941	405 (83.51%)
	M1	42 (11.05%)	19 (11.88%)	61 (10.89%)		80 (16.49%)
TNM_N	N0	204 (51.78%)	95 (56.55%)	314 (53.68%)	0.576	326 (58.53%)
	N1/2/3	190 (48.22%)	73 (43.45%)	271 (46.32%)		231 (41.47%)
TNM_T	T1/2	36 (9.14%)	19 (11.31%)	61 (10.43%)	0.690	103 (18.49%)
	T3/4	358 (90.86%)	149 (88.69%)	524 (89.57%)		454 (81.51%)
OS	0	261 (66.24%)	110 (65.48%)	385 (66.49%)	0.970	412 (77.30%)
	1	133 (33.76%)	58 (34.52%)	194 (33.51%)		121 (22.70%)
OS.time	≤2	232 (58.88%)	100 (59.52%)	334 (57.69%)	0.884	266 (49.91%)
	>2	162 (41.12%)	68 (40.48%)	245 (42.31%)		267 (50.09%)

MMR, defective mismatch repair; OS, overall survival. NA, Not applicable.

### Construction and validation of the DRG prognostic model in COAD patients

**Figure 1:**
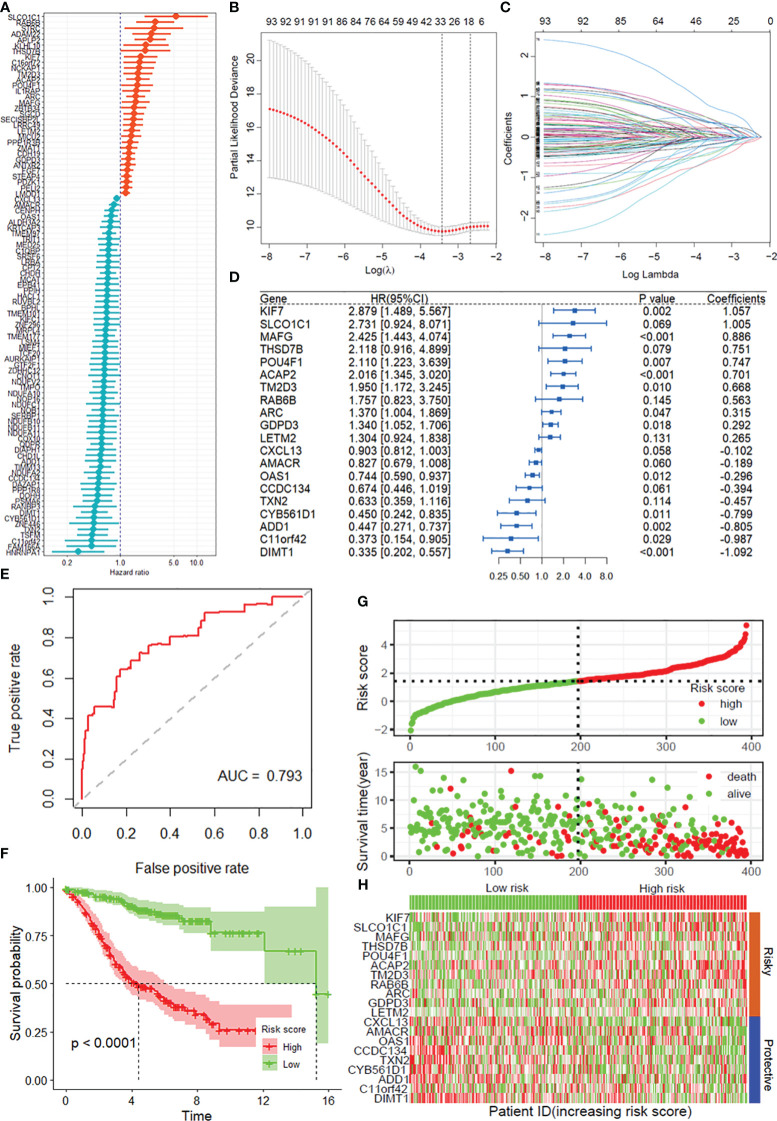
Construction of the DRG prognostic model of COAD. **(A)** Univariate Cox regression analysis for the selection of DRGs correlated with the overall survival (OS) of COAD patients. **(B, C)** LASSO-penalized Cox analysis revealed 20 DRGs related to overall survival. **(D)** Forest plot showing the multivariate Cox regression analysis of 20 DRGs. **(E)** ROC curves for 5-year OS in the training set. **(F)** Kaplan–Meier curve of overall survival in the training group. **(G)** Risk score distribution and survival status of the training group. **(H)** Heatmap showing the expression of genes in 20 DRGs in the training group. DRGs, disulfidptosis-related genes; ROC, receiver operating characteristic; COAD, colon adenocarcinoma.

A total of 808 DRGs were screened with the criteria of |normZ values| >2 and *P*-value <0.05 based on the CRISPR–Cas9 screenings (**Supplementary Figure S1**). Using univariate Cox regression analysis, 95 prognosis-related DRGs were identified based on the GEO training set (**Figure 1A**). Consequently, LASSO-penalized Cox analysis further identified 20 DRGs for multivariate analysis (**Figures 1B, C**). The multivariate Cox proportional hazard model was built stepwise using the likelihood-ratio forward method to reach the highest significance. A total of 20 DRGs were further screened to construct a risk model to assess the prognostic risk of COAD patients: risk score = (1.057 × KIF7 Exp) + (1.005 × SLCO1C1 Exp) + (0.886 × MAFG Exp) + (0.751 × THSD7B Exp) + (0.747 × POU4F1 Exp) + (0.701 × ACAP2 Exp) + (0.668 × TM2D3 Exp) + (0.563 × RAB6B Exp) + (0.315 × ARC Exp) + (0.292 × GDPD3 Exp) + (0.265 × LETM2 Exp) + (-0.102 × CXCL13 Exp) + (-0.189 × AMACR Exp) + (-0.296 × OAS1 Exp) + (-0.394 × CCDC134 Exp) + (-0.457 × TXN2 Exp) + (-0.799 × CYB561D1 Exp) + (-0.805 × ADD1 Exp) + (-0.987 × C11of42 Exp) + (-1.092 × DIMT1 Exp) (**Figure 1D**). ROC curves demonstrated that the risk score serves as a significant predictor of the OS of COAD patients, with AUCs greater than 0.765 at 1–5 years (**Figure 1E**). K–M survival analysis indicated that the low-risk group had a significantly favorable overall survival for COAD patients (**Figure 1F**). The samples in the training set were classified into low-risk and high-risk groups based on the median value of the risk score (**Figure 1G**). The distribution of risk scores between the low-risk and high-risk groups and the survival status and survival time of patients in the two different risk groups are depicted. The relative expression of the 20 DRGs for each patient is shown in a heatmap (**Figure 1H**).

### Prognostic value of the DRG model signature in the training cohort and validation cohort

**Figure 2:**
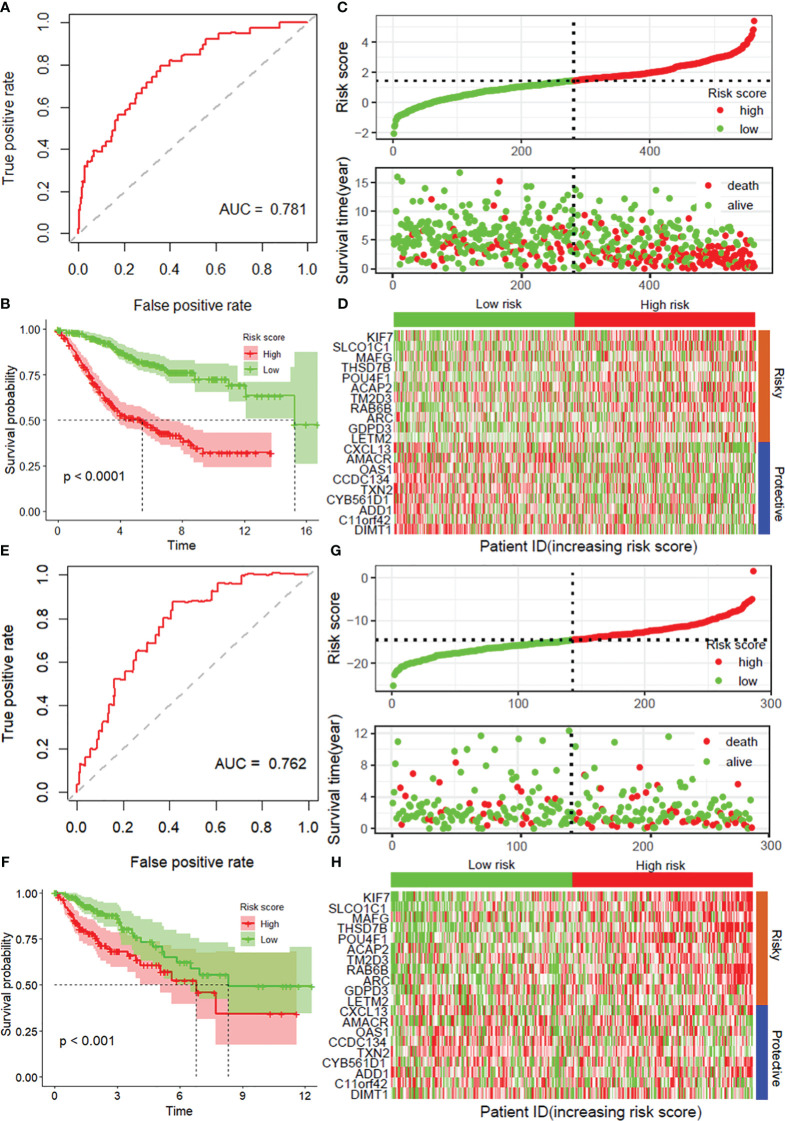
Validation of the prognostic model with 20 DRGs constructed from the training dataset. ROC curves for overall survival in the entire GSE39582 **(A)** and TCGA-COAD **(E)** datasets. K−M curves of overall survival in the entire GSE39582 **(B)** and TCGA-COAD **(F)** datasets. Risk score distribution and survival status in the entire GSE39582 dataset **(C)** and external validation dataset **(G)**. Heatmaps showing the expression of these 20 disulfidptosis-related risk genes between the low-risk group and the high-risk group in the entire GSE39582 dataset **(D)** and external validation dataset **(H)**. DRGs, disulfidptosis-related genes; ROC, receiver operating characteristic; COAD, colon adenocarcinoma.

According to the median risk score, the patients in the internal testing dataset and the entire GSE39582 dataset were divided into high-risk and low-risk groups. The patients in the low-risk group in both datasets had a significantly longer OS than did those in the high-risk group (*p* < 0.0001), with AUC values of 0.760 and 0.781, respectively (**Supplementary Figures S2A, B**, **Figures 2A, B**). According to the distribution of risk scores, the number of deaths in the high-risk group was significantly greater than that in the low-risk group (**Supplementary Figure S2C**, **Figure 2C**). The heatmap showed the differential expression of these 20 disulfidptosis-related risk genes between the low-risk group and the high-risk group (**Supplementary Figure S2D**, **Figure 2D**). The signature in the independent validation set also yielded the same result (**Figures 2E–H**).

### DRG risk score is independent of clinical features

**Figure 3:**
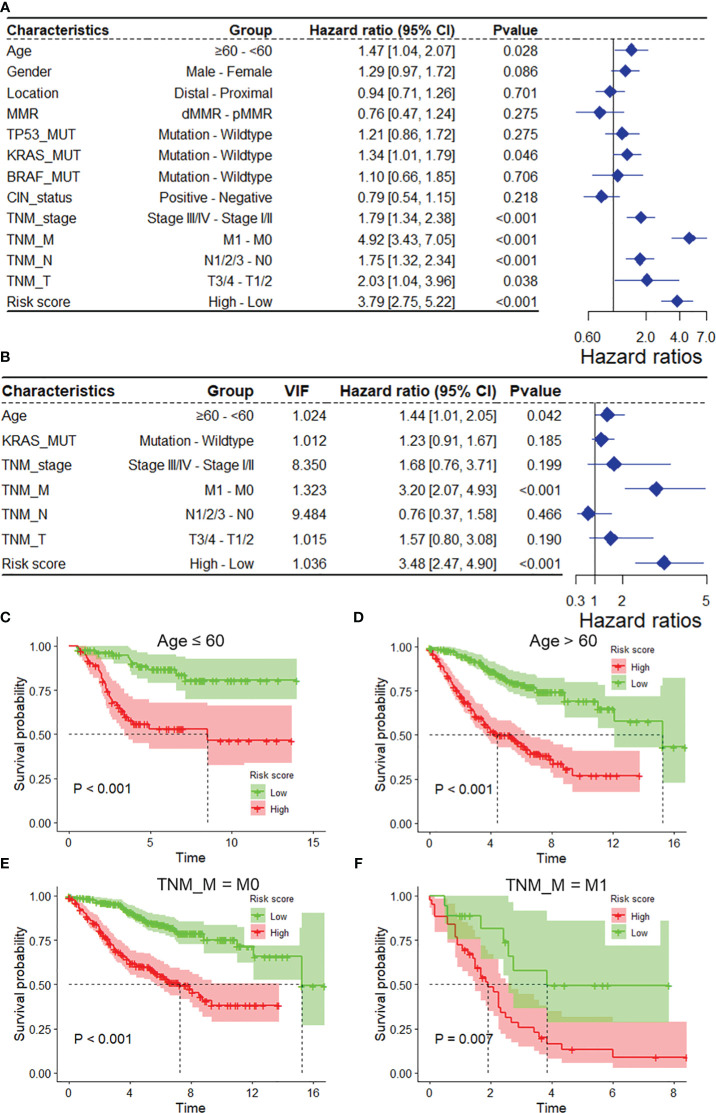
The DRG risk score was an independent prognostic factor for overall survival in the entire GSE39582 dataset. Univariate **(A)** and multivariate **(B)** Cox regression analyses of the risk score and clinicopathological features for overall survival in the entire GSE39582 dataset. **(C, D)** Kaplan−Meier analysis of overall survival stratified by the patients’ age. **(E, F)** Kaplan−Meier analysis of overall survival stratified by TNM_M stage. DRGs, disulfidptosis-related genes; VIF, variance inflation factor.

As depicted in **Supplementary Table S1**, the mitophagy risk score was related to several clinicopathological features in the GSE39582 dataset, including MMR, TNM_stage, TNM_M, TNM_N, and TNM_T. To assess whether the risk score is an independent indicator in COAD patients, the effect of each clinicopathological feature on OS was analyzed by univariate Cox regression (**Figure 3A**). As shown in **Figure 3B**, after multicollinearity test and multivariable adjustment, the risk score remained a powerful and independent factor in the GSE39582 dataset. Moreover, the risk score was verified as an independent factor based on the TCGA-COAD dataset (**Supplementary Figures S3A, B**). The discrepancies in OS stratified by M_stage and age were analyzed between the low-risk and high-risk groups in the GSE39582 and TCGA-COAD datasets. According to the subgroups classified by age and M stage, the OS of the low-risk score group was superior to that of the high-risk group (**Figures 3C–F**, **Supplementary Figures S3C, D**).

**Figure 4:**
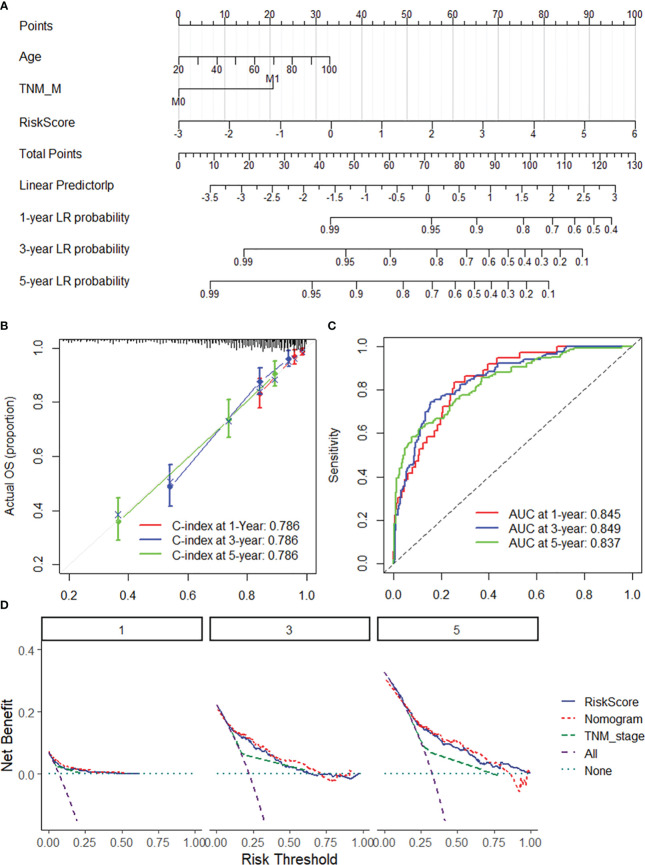
Nomogram for predicting the 1-y, 3-, and 5-year overall survival of COAD patients. **(A)** The nomogram consists of the 20-DRG risk scores and 12 clinical indicators based on the entire GSE39582 dataset. The points from these variables are combined, and the locations of the total points are determined. The total points projected on the bottom scales indicate the probabilities of 1-, 3-, and 5-year overall survival. **(B)** 1/3/5-year calibration plot validating the accuracy of the prognostic nomogram based on the GSE39582 dataset. **(C)** Kaplan–Meier curve of overall survival for the score calculated from the nomogram in the GSE39582 dataset. **(D)** DCA curves of the nomogram; the 20-DRG risk score and the TNM stage system for the prediction of OS prognosis at the 1-, 3-, and 5-year time points in the GSE39582 dataset. DRGs, disulfidptosis-related genes; COAD, colon adenocarcinoma; OS, overall survival; DCA, decision curve analysis.

To ensure the robustness and practicability of the 20-DRG prognostic model, a prognostic nomogram for predicting overall survival in COAD patients was established using the GSE31210 and TCGA-COAD datasets (**Figure 4A**, **Supplementary Figure S4A**). Major clinicopathological features and risk scores were included in the nomogram. The nomogram was internally validated by computing the bootstrap C-index (≥0.785 both in the GSE31210 and TCGA-COAD datasets) and a calibration plot (**Figure 4B**, **Supplementary Figure S4B**). The ROC curve confirmed that the score calculated based on the nomogram was highly predictive of overall survival, with AUCs of 0.845 and 0.862 at 1 year in the GSE31210 and TCGA-COAD cohorts, respectively (**Figure 4C**, **Supplementary Figure S4C**). The DCA for the nomogram is presented in **Figure 4D**. The nomogram provided a better net benefit than did the “treat-all” or “treat-none” schemes and the current TNM staging system.

### DRG risk score correlated with immune cell infiltration

**Figure 5:**
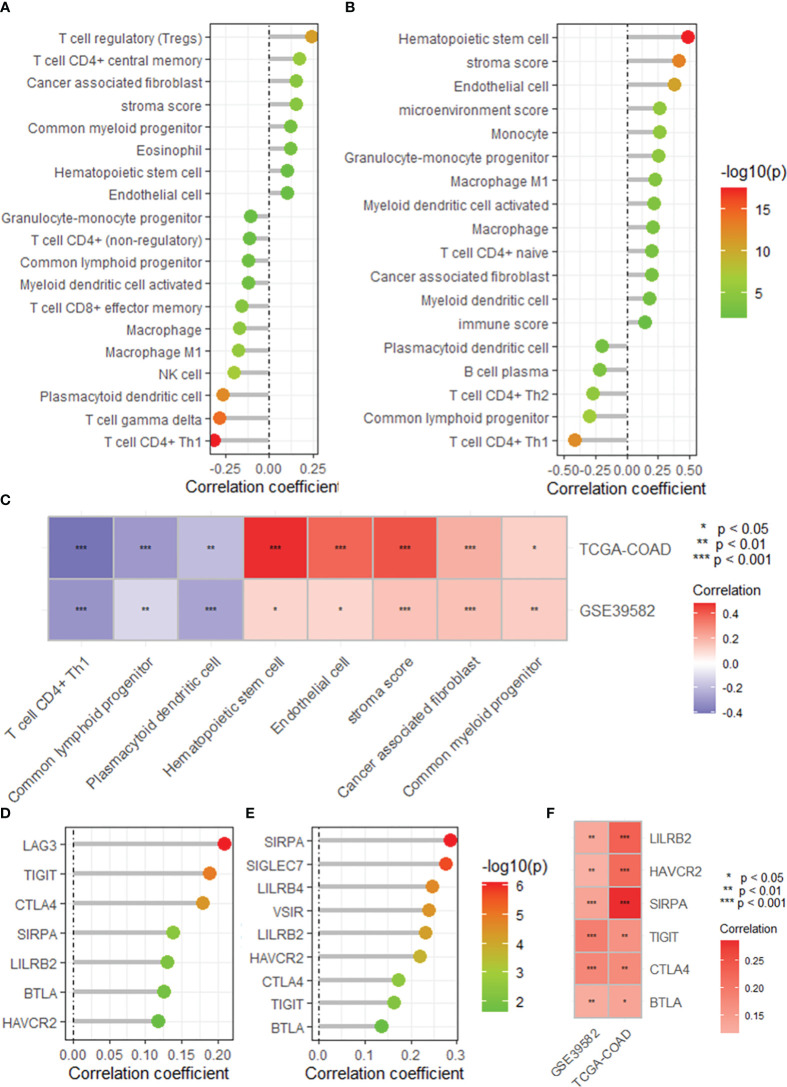
The DRG risk score correlated with immune cell infiltration and immune checkpoint gene expression. Lollipop plots showing the correlation between the risk score and the infiltration of immune cells calculated by the XCELL algorithm based on the GSE395852 **(A)** and TCGA-COAD **(B)** datasets. **(C)** Heatmap showing the intersection of the correlated immune cell types in the TCGA-COAD and GSE39582 datasets. Lollipop plots showing the correlation between the risk score and the expression of immune checkpoint genes based on the GSE395852 **(D)** and TCGA-COAD **(E)** datasets. **(F)** Heatmap showing the intersection of the correlated immune cell types in the TCGA-COAD and GSE39582 datasets. DRGs, disulfidptosis-related genes; COAD, colon adenocarcinoma.

The single-sample gene set enrichment analysis package “XCELL” was used to quantify the infiltration of 24 immune cell types, and Spearman correlation analysis was used to assess the correlation of immune cell infiltration with the DRG risk score. The results revealed that the risk score was significantly correlated with the infiltration of multiple immune cell types in both the GEO39582 and TCGA-COAD datasets (**Figures 5A, B**). Specifically, we found a significant negative correlation between risk scores and T cells and the infiltration of CD4+ T cells (Th1), common lymphoid dendritic progenitors, and plasmacytoid dendritic cells in both datasets (**Figure 5C**). Additionally, the risk score was positively correlated with the infiltration of hematopoietic stem cells, endothelial cells, stroma score, cancer-associated fibroblasts, and common myeloid progenitors (**Figure 5C**). In addition, a correlation analysis revealed that the risk score was positively correlated with the expression of several immune checkpoint genes, mainly LILRB2, HAVCR2, SIRPA, TIGIT, CTLA4, and BTLA, in both datasets (**Figures 5D–F**). As shown in **Supplementary Figure S5**, we found that the risk score was significantly correlated with the expression of multiple immune regulatory genes and the sensitivity to multiple antitumor drugs.

### DRG risk score correlated with cancer progression

**Figure 6:**
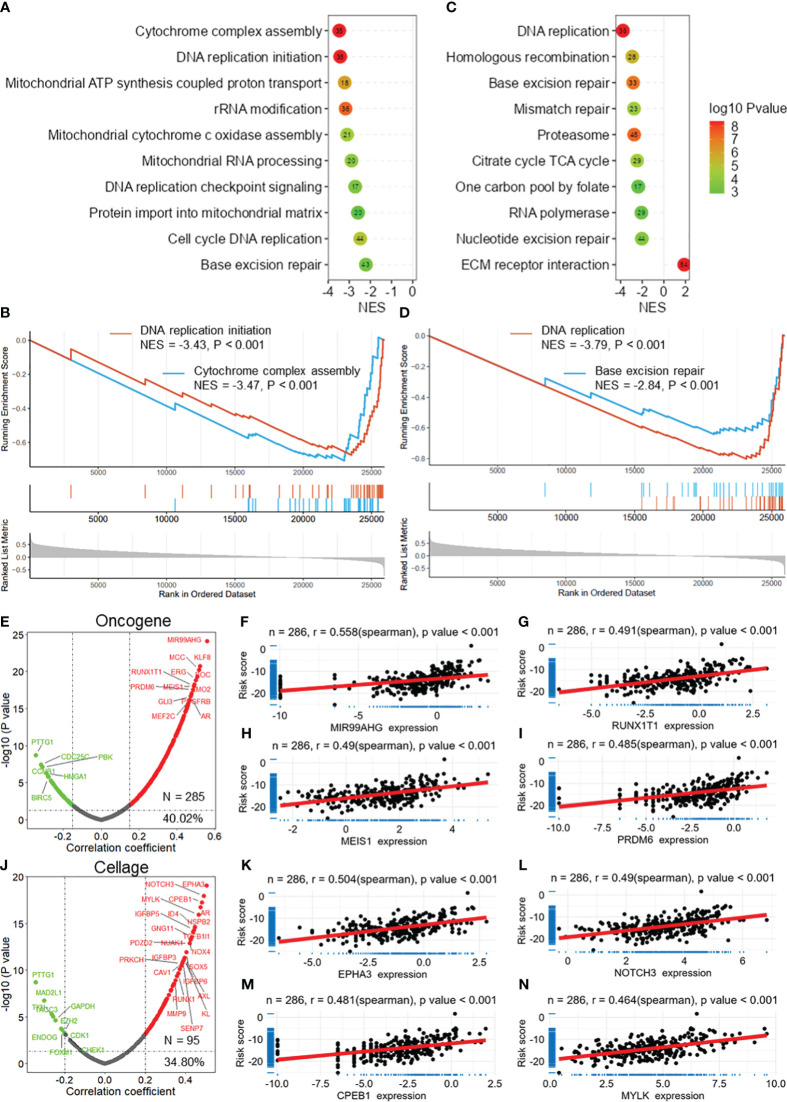
DRG risk score correlated with cancer progression. **(A)** Lollipop plots showing the results of GSEA for the biological processes associated with the DRG risk score. **(B)** GSEA plots showing that the risk score is related to DNA replication initiation and cytochrome complex assembly. **(C)** Lollipop plots showing the results of GSEA of the KEGG pathways associated with the DRG risk score. **(D)** GSEA plots showing that the risk score is related to DNA replication and base excision repair. **(E)** The volcano plot shows the results of the correlation analysis between the DRG risk score and oncogenes extracted from the ONGene database. Scatter plots showing the correlation between the risk score and MIR99AHG **(F)**, RUNX1T1 **(G)**, MEIS1 **(H)**, and PRDM6 **(I)**. **(J)** The volcano plot shows the results of the correlation analysis between the DRG risk score and cell senescence-related genes extracted from the ONGene database. Scatter plot showing the correlations between the risk score and EPHA3 **(K)**, NOTCH3 **(L)**, CPEB1 **(M)**, and MYLK **(N)** expression. GSEA, gene set enrichment analysis; DRGs, disulfidptosis-related genes.

GSEA revealed that the DRG risk score was significantly correlated with several vital cancer-related biological processes (**Figure 6A**), mainly cytochrome complex assembly (NES = -3.47, **Figure 6B**), DNA replication initiation (NES = -3.43, **Figure 6B**), mitochondrial cytochrome c oxidase assembly, cell cycle DNA replication, and base excision repair. In addition, the risk score was related to several important KEGG pathways (**Figure 6C**), including DNA replication (NES = -3.79, **Figure 6D**), base excision repair (NES = -2.849, **Figure 6D**), mismatch repair, and ECM receptor interaction. Moreover, the correlation analysis demonstrated that the DRG risk score was significantly positively correlated with multiple oncogenes (*N* = 285, 40.2%, **Figure 6E**), including MIR99AHG (*r* = 0.558, **Figure 6F**), RUNX1T1 (*r* = 0.491, **Figure 6G**), MEIS1 (*r* = 0.490, **Figure 6H**), and PRDM6 (*r* = 0.485, **Figure 5I**). In addition, we found that the DRG risk score was positively correlated with many cell senescence-related genes (*N* = 95, 34.80%, **Figure 6J**), including EPHA3 (*r* = 0.504, **Figure 6K**), NOTCH3 (*r* = 0.490, **Figure 6L**), CPEB1 (*r* = 0.481, **Figure 6M**), and MYLK (*r* = 0.464, **Figure 6N**). These results revealed that the DRG risk score was correlated with multiple oncogenes and cell senescence-related genes as well as several cancer-related biological bioprocesses and KEGG pathways.

### POU4F1 is highly expressed in COAD and is related to cancer progression

**Figure 7:**
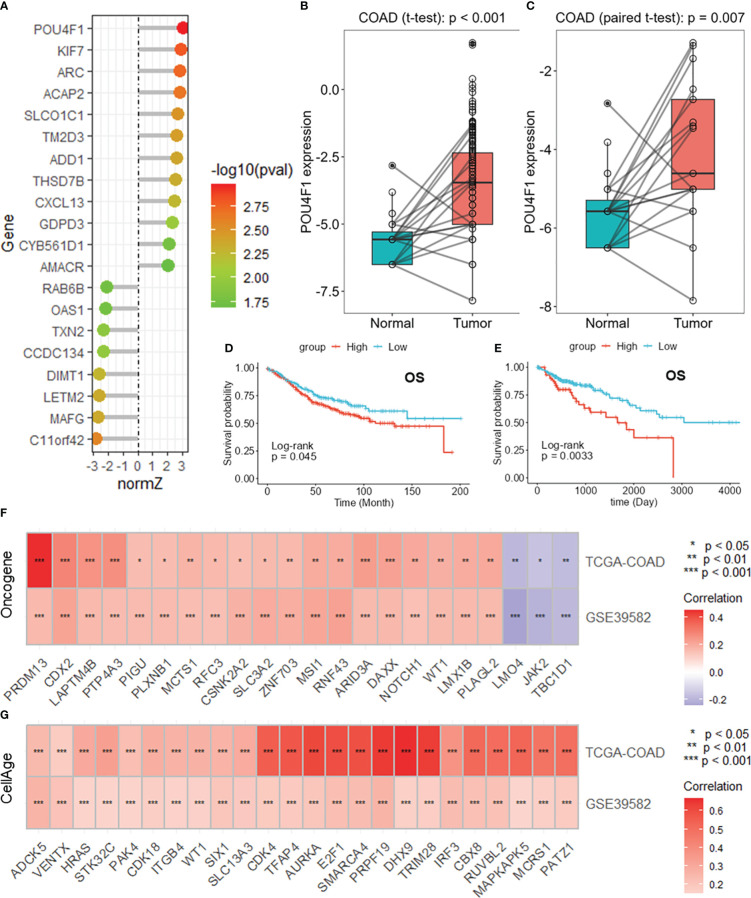
POU4F1 is highly expressed in COAD and is related to cancer progression. **(A)** Lollipop plot showing the normalized Z scores of the DRGs in the risk model. Boxplots showing the differential expression of POU4F1 in the whole TCGA-COAD dataset **(B)** and paired samples **(C)**. Survival analysis of POU4F1 for overall survival in the GSE39582 **(D)** and TCGA-COAD **(E)** datasets. Heatmaps showing the intersection of the oncogenes **(F)** and cell senescence-related genes **(G)** correlated with POU4F1 in the GSE39582 and TCGA-COAD datasets. DRGs, disulfidptosis-related genes; COAD, colon adenocarcinoma. * p<0.05,** p<0.01,***p<0.001

Among these DRGs in the constructed risk model, POU4F1 had the highest normalized Z score (**Figure 7A**). POU4F1 expression was greater in COAD tumor tissues than in normal tissues in the TCGA-COAD dataset (**Figure 7B**). Additionally, an increased expression of POU4F1 was detected in paired normal tissue specimens (**Figure 7C**). A survival analysis revealed that patients with lower POU4F1 expression had a longer overall survival in both the GSE395852 (**Figure 7D**) and TCGA-COAD (**Figure 7E**) datasets. When considering disease-specific survival and disease-free survival, a better prognosis was found for patients with low POU4F1 expression (**Supplementary Figures S6A, B**). A further correlation analysis revealed that POU4F1 expression was significantly correlated with multiple oncogenes in both the TCGA-COAD and GSE39582 datasets (**Figure 7F**, **Supplementary Figures S6C, D**), indicating that POU4F1 may serve as an oncogene in COAD. Additionally, POU4F1 expression was positively correlated with multiple cell senescence-related genes in both the TCGA-COAD and GSE39582 datasets (**Figure 7G**, **Supplementary Figures S6E, F**).

### POU4F1 promotes cell proliferation, migration, and disulfidptosis in COAD

**Figure 8:**
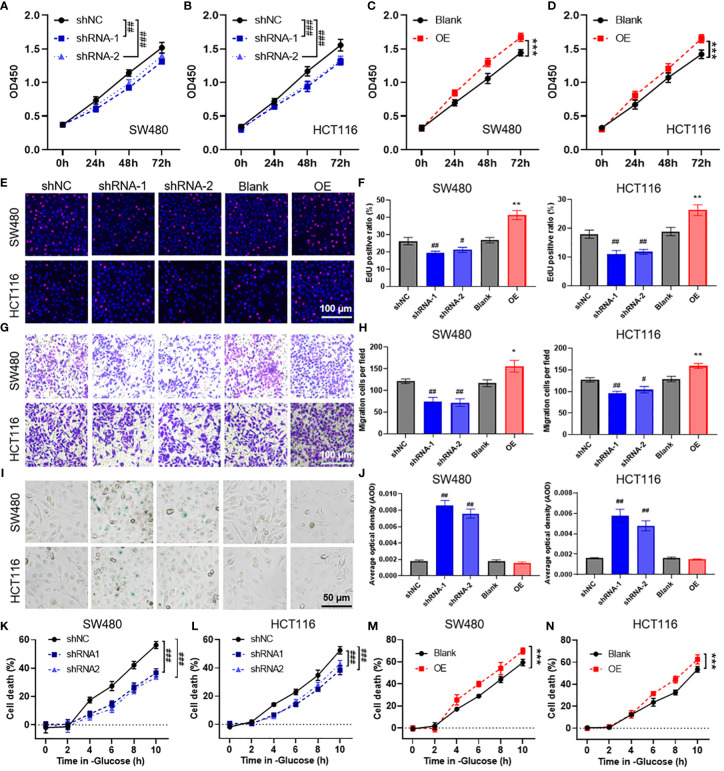
POU4F1 promotes cell proliferation, migration, and disulfidptosis in colon adenocarcinoma (COAD). **(A–D)** A CCK-8 assay was used to measure the effect of POU4F1 knockdown or overexpression in SW480 and HCT116 cells. Representative images **(E)** and the quantified results **(F)** of the EdU cell proliferation assay for COAD cells with POU4F1 knockdown or knockdown. Representative images **(G)** and the quantified results **(H)** of the Transwell cell migration assay for COAD cells with POU4F1 knockdown or knockdown. Representative images **(I)** and the quantified results **(J)** of the β-galactosidase staining assay for COAD cells with POU4F1 knockdown or knockdown. **(K–N)** A CCK-8 assay was used to measure the cell death induced by glucose deprivation in COAD cells with POU4F1 knockdown or knockdown. Compare with shNC, # p<0.05, ## p<0.01, ### p<0.001; compare with Blank, * p<0.05, ** p<0.01, *** p<0.001.

To evaluate the biological function of POU4F1 in COAD cells, we constructed shRNA plasmids to knock down POU4F1 and a plasmid to overexpress POU4F1 (**Supplementary Figure S7**). The CCK-8 assay demonstrated that POU4F1 knockdown significantly inhibited the proliferation of SW480 (**Figure 8A**) and HCT116 (**Figure 8B**) cells. Conversely, POU4F1 overexpression significantly promoted proliferation (**Figures 8C, D**). Additionally, the EdU assay revealed that POU4F1 knockdown attenuated COAD cell proliferation, while POU4F1 overexpression increased proliferation (**Figures 8E, F**). The Transwell migration assay indicated that POU4F1 knockdown significantly reduced the number of migrated cells, while POU4F1 overexpression significantly increased the number of migrated cells (**Figures 8G, H**). During cell senescence, the β-galactosidase (β-gal) staining assay demonstrated that POU4F1 knockdown significantly promoted β-gal expression (**Figures 8I, J**). To further evaluate the synergistic role of POU4F1 in disulfidptosis, we used a glucose-deprived medium to culture COAD cells. The results of the CCK-8 assay revealed that POU4F1 knockdown significantly attenuated cell death induced by glucose deprivation, while POU4F1 overexpression significantly amplified cell death (**Figures 8K–N**).

## Discussion

Colorectal adenocarcinoma (COAD) has emerged as a significant global medical challenge attributed to environmental factors and genetic mutations and has an alarming increase in younger patients. Advances in gene sequencing technology and the accessibility of public genetic databases have enabled us to predict COAD prognosis by quantifying molecular prognostic markers and constructing prognostic models (16). Disulfidptosis, which is a newly recognized form of regulated cell death in cancers with high SLC7A11 expression under glucose starvation conditions, is a novel therapeutic strategy for treating malignant tumors (7, 8). In this study, we developed a prognosis prediction model based on disulfidptosis-related genes using LASSO and Cox regression analyses. Subsequently, we identified a key gene in this model, namely, POU4F1, for further functional analysis.

Risk prediction models have been developed for various cancers, including cervical cancer, bladder cancer, and colorectal cancer, based on disulfidptosis-related genes (DRGs). For colorectal cancer specifically, a previous study constructed a risk prediction model based on genes, achieving an AUC of 0.567 at 1 year (17). Another study developed a model based on four lncRNAs, with an AUC of 0.679 at 1 year (18). In comparison to these studies, our research established a risk prediction model with an AUC of 0.793 using DRGs based on DRGs obtained from CRISPR Cas9 screening results that Gan et al. published. The superiority of our model was further validated using internal testing and external validation sets, which achieved AUC values of 0.781 and 0.762, respectively. The robustness and practicality of our model were assessed using a nomogram, which demonstrated an improved prediction accuracy with an AUC of 0.845 based on the risk score. These results highlight the favorable predictive accuracy and practical value of our DRG prognostic model.

The tumor microenvironment (TME) has garnered significant attention due to its crucial role in tumor immunosuppression, distant metastasis, and drug resistance (19). The TME is primarily composed of tumor cells, infiltrating immune cells, cancer-related stromal cells, endothelial cells, and other components (20, 21). Among the various stromal cells within the TME, cancer-related fibroblasts (CAFs) are recognized as key contributors that exhibit tumor-promoting effects and participate in multiple stages of tumor development through various pathways (22, 23). Tumor endothelial cells, another important type of stromal cell in the TME, have been reported to release “angiocrine factors” that promote tumor progression (24). Through a correlation analysis, we found that the risk score derived from our model was positively correlated with the stromal score and the infiltration of endothelial cells and CAFs. Additionally, the risk score was correlated with the expression of several immune checkpoint molecules, including BTLA, CTLA4, and SIRPA. Immune checkpoint genes regulate the immune system by either stimulating or suppressing immune responses, and this regulatory mechanism is widely observed in tumors under physiological conditions (25). Gene set enrichment analysis (GSEA) revealed potential biological processes associated with the risk score. Our findings indicate that the risk score is correlated with DNA replication, cytochrome complex assembly, and base excision repair. Moreover, we observed a positive correlation between the risk score and the expression of multiple oncogenes and cell senescence-associated genes. Cellular aging, characterized by permanent cell cycle arrest, is characterized by various physiological and pathological processes, such as tissue remodeling, injury, cancer, and aging. While cellular aging acts as an effective barrier to prevent tumors, there are instances where aged cells can support tumor progression (26). These results suggest that our risk prediction model based on DRGs has potential as an indicator for predicting immune microenvironment homeostasis, evaluating immune checkpoint blockade therapy, and assessing the biological functional status of tumors. Although this study proposes a prognostic prediction model based on DRGs and preliminarily validates the role of POU4F1 in COAD, these findings remain hypothetical and require additional experimental validation and clinical research to confirm their effectiveness in practical clinical applications.

Among the 20 DRGs included in our risk prediction model, we focused on POU4F1, which demonstrated the highest normalized Z score based on CRISPR screening. Our aim was to investigate its role in regulating biological functions and cell death in COAD cells. Previous studies have identified POU4F1 as a factor that induces resistance to trastuzumab in breast cancer cells by mediating the ERK1/2 pathway (27). As a transcription factor, POU4F1 has been shown to transcribe and regulate the expression of MEK in melanoma, thereby reactivating the MAPK pathway and leading to resistance against BRAF inhibitors (28). Our findings indicate that POU4F1 may act as an oncogene due to its upregulation in COAD tumors and its positive correlation with the expression of oncogenes. Furthermore, survival analysis revealed that a high POU4F1 expression was associated with a poor prognosis in COAD patients. Further *in vivo* assays indicated that POU4F1 knockdown significantly attenuated cell proliferation and migration while increasing cell senescence in COAD cells. Our research highlights the nuanced roles of SLC7A11 and POU4F1 in COAD, where SLC7A11 overexpression may inhibit metastasis, in contrast with the ability of POU4F1 to facilitate tumor growth and migration, suggesting that gene functions vary significantly across cancers due to unique genetic and epigenetic landscapes (29). We further evaluated the regulatory effect of the DRGs on disulfidptosis by culturing the cells in a glucose-deprived medium. The results demonstrated that POU4F1 knockdown inhibited glucose deprivation-induced cell death, while POU4F1 overexpression promoted cell death. These results revealed that POU4F1 has important effects on the proliferation, migration, and senescence of COAD cells as well as disulfidptosis. In summary, our study revealed and validated a risk prediction model based on DRGs in COAD patients. Furthermore, we have provided evidence that POU4F1 promotes cell proliferation, migration, and disulfidptosis in COAD.

In conclusion, our study revealed and verified a risk prediction model based on disulfidptosis-related genes (DRGs) in COAD patients. The risk score is related to immune microenvironment homeostasis, expression of immune checkpoints, and tumor biological functions. POU4F1, a crucial component of this model, has been confirmed to promote cell proliferation, migration, and disulfidptosis in COAD cells. This prognostic model not only enhances our understanding of COAD progression mechanisms but also provides a new tool for the stratified management of colorectal cancer patients, allowing clinicians to more accurately predict patient prognosis and formulate personalized treatment plans, thereby improving treatment outcomes and patient survival rates.

## Data availability statement

The datasets presented in this study can be found in online repositories. The names of the repository/repositories and accession number(s) can be found in the article/**Supplementary Material**.

## Ethics statement

Ethical approval was not required for the studies on animals in accordance with the local legislation and institutional requirements because only commercially available established cell lines were used.

## Author contributions

ML: Conceptualization, Data curation, Formal analysis, Investigation, Methodology, Software, Writing – original draft, Writing – review & editing, Funding acquisition. JW: Conceptualization, Data curation, Methodology, Software, Writing – original draft. YZ: Data curation, Methodology, Writing – original draft, Formal analysis, Investigation, Project administration, Resources, Visualization. CL: Formal analysis, Supervision, Writing – review & editing. JM: Writing – original draft, Methodology, Software. XM: Writing – review & editing, Methodology, Software. ZY: Writing – review & editing, Methodology, Software. CC: Data curation, Writing – review & editing, Software. KT: Writing – original draft, Methodology, Software. PZ: Writing – review & editing. QH: Formal analysis, Writing – original draft. JS: Data curation, Funding acquisition, Methodology, Writing – review & editing. JG: Data curation, Funding acquisition, Methodology, Writing – review & editing, Project administration, Resources, Supervision, Validation, Visualization. SW: Funding acquisition, Methodology, Writing – review & editing, Data curation, Formal analysis, Investigation, Validation, Writing – original draft.

## References

[1] Sharma R Abbasi-Kangevari M Abd-Rabu R Abidi H Abu-Gharbieh E Acuna JM. Global, regional, and national burden of colorectal cancer and its risk factors, 1990-2019: a systematic analysis for the Global Burden of Disease Study 2019. Lancet Gastroenterol Hepatol. (2022) 7:627–47. doi: 10.1016/s2468-1253(22)00044-9 PMC9192760 35397795

[2] Sung H Ferlay J Siegel RL Laversanne M Soerjomataram I Jemal A. Global cancer statistics 2020: GLOBOCAN estimates of incidence and mortality worldwide for 36 cancers in 185 countries. CA Cancer J Clin. (2021) 71:209–49. doi: 10.3322/caac.21660 33538338

[3] Siegel RL Wagle NS Cercek A Smith RA Jemal A. Colorectal cancer statistics, 2023. CA Cancer J Clin. (2023) 73:233–54. doi: 10.3322/caac.21772 36856579

[4] Lui RN Tsoi KKF Ho JMW Lo CM Chan FCH Kyaw MH. Global increasing incidence of young-onset colorectal cancer across 5 continents: A joinpoint regression analysis of 1,922,167 cases. Cancer Epidemiol Biomarkers Prev. (2019) 28:1275–82. doi: 10.1158/1055-9965.Epi-18-1111 31113868

[5] Liu X Nie L Zhang Y Yan Y Wang C Colic M. Actin cytoskeleton vulnerability to disulfide stress mediates disulfidptosis. Nat Cell Biol. (2023) 25:404–14. doi: 10.1038/s41556-023-01091-2 PMC10027392 36747082

[6] Liu X Olszewski K Zhang Y Lim EW Shi J Zhang X. Cystine transporter regulation of pentose phosphate pathway dependency and disulfide stress exposes a targetable metabolic vulnerability in cancer. Nat Cell Biol. (2020) 22:476–86. doi: 10.1038/s41556-020-0496-x PMC7194135 32231310

[7] Zheng P Zhou C Ding Y Duan S. Disulfidptosis: a new target for metabolic cancer therapy. J Exp Clin Cancer Res. (2023) 42:103. doi: 10.1186/s13046-023-02675-4 37101248 PMC10134647

[8] Yang L Liu J Li S Liu X Zheng F Xu S. Based on disulfidptosis, revealing the prognostic and immunological characteristics of renal cell carcinoma with tumor thrombus of vena cava and identifying potential therapeutic target AJAP1. J Cancer Res Clin Oncol. (2023) 149(12):9787–804. doi: 10.1007/s00432-023-04877-x PMC11796798 37247081

[9] Qi C Ma J Sun J Wu X Ding J. The role of molecular subtypes and immune infiltration characteristics based on disulfidptosis-associated genes in lung adenocarcinoma. Aging (Albany NY). (2023) 15:5075–95. doi: 10.18632/aging.204782 PMC10292876 37315289

[10] Zhao M Kim P Mitra R Zhao J Zhao Z. TSGene 2.0: an updated literature-based knowledgebase for tumor suppressor genes. Nucleic Acids Res. (2016) 44:D1023–1031. doi: 10.1093/nar/gkv1268 PMC4702895 26590405

[11] Charoentong P Finotello F Angelova M Mayer C Efremova M Rieder D. Pan-cancer immunogenomic analyses reveal genotype-immunophenotype relationships and predictors of response to checkpoint blockade. Cell Rep. (2017) 18:248–62. doi: 10.1016/j.celrep.2016.12.019 28052254

[12] Aran D Hu Z Butte AJ. xCell: digitally portraying the tissue cellular heterogeneity landscape. Genome Biol. (2017) 18:220. doi: 10.1186/s13059-017-1349-1 29141660 PMC5688663

[13] Jiang P Gu S Pan D Fu J Sahu A Hu X. Signatures of T cell dysfunction and exclusion predict cancer immunotherapy response. Nat Med. (2018) 24:1550–8. doi: 10.1038/s41591-018-0136-1 PMC6487502 30127393

[14] Yang W Soares J Greninger P Edelman EJ Lightfoot H Forbes S. Genomics of Drug Sensitivity in Cancer (GDSC): a resource for therapeutic biomarker discovery in cancer cells. Nucleic Acids Res. (2013) 41:D955–961. doi: 10.1093/nar/gks1111 PMC3531057 23180760

[15] Maeser D Gruener RF Huang RS. oncoPredict: an R package for predicting in vivo or cancer patient drug response and biomarkers from cell line screening data. Brief Bioinform. (2021) 22(6):bbab260. doi: 10.1093/bib/bbab260 PMC8574972 34260682

[16] Wu M Li X Liu R Yuan H Liu W Liu Z. Development and validation of a metastasis-related Gene Signature for predicting the Overall Survival in patients with Pancreatic Ductal Adenocarcinoma. J Cancer. (2020) 11:6299–318. doi: 10.7150/jca.47629 PMC7532518 33033514

[17] Hu G Yao H Wei Z Li L Yu Z Li J. A bioinformatics approach to identify a disulfidptosis-related gene signature for prognostic implication in colon adenocarcinoma. Sci Rep. (2023) 13:12403. doi: 10.1038/s41598-023-39563-y 37524774 PMC10390519

[18] Chen H Yang W Li Y Ma L Ji Z. Leveraging a disulfidptosis-based signature to improve the survival and drug sensitivity of bladder cancer patients. Front Immunol. (2023) 14:1198878. doi: 10.3389/fimmu.2023.1198878 37325625 PMC10266281

[19] Chen F Zhuang XQ Lin LY Yu PF Wang Y Shi YF. New horizons in tumor microenvironment biology: challenges and opportunities. BMC Med. (2015) 13:45. doi: 10.1186/s12916-015-0278-7 PMC4350882 25857315

[20] Giraldo NA Sanchez-Salas R Peske JD Vano Y Becht E Petitprez F. The clinical role of the TME in solid cancer. Brit J Cancer. (2019) 120:45–53. doi: 10.1038/s41416-018-0327-z 30413828 PMC6325164

[21] Li HC Fan XL Houghton J. Tumor microenvironment: The role of the tumor stroma in cancer. J Cell Biochem. (2007) 101:805–15. doi: 10.1002/jcb.21159 17226777

[22] Fiori ME Di Franco S Villanova L Bianca P Stassi G De Maria R. Cancer-associated fibroblasts as abettors of tumor progression at the crossroads of EMT and therapy resistance. Mol Cancer. (2019) 18(1):70. doi: 10.1186/s12943-019-0994-2 PMC6441236 30927908

[23] Hinshaw DC Shevde LA. The tumor microenvironment innately modulates cancer progression. Cancer Res. (2019) 79:4557–66. doi: 10.1158/0008-5472.Can-18-3962 PMC6744958 31350295

[24] Maishi N Hida K. Tumor endothelial cells accelerate tumor metastasis. Cancer Sci. (2017) 108:1921–6. doi: 10.1111/cas.13336 PMC5623747 28763139

[25] Morad G Helmink BA Sharma P Wargo JA. Hallmarks of response, resistance, and toxicity to immune checkpoint blockade. Cell. (2021) 184:5309–37. doi: 10.1016/j.cell.2021.09.020 PMC8767569 34624224

[26] Calcinotto A Kohli J Zagato E Pellegrini L Demaria M Alimonti A. Cellular senescence: aging, cancer, and injury. Physiol Rev. (2019) 99:1047–78. doi: 10.1152/physrev.00020.2018 30648461

[27] Wu D Jia HY Wei N Li SJ. POU4F1 confers trastuzumab resistance in HER2-positive breast cancer through regulating ERK1/2 signaling pathway. Biochem Biophys Res Commun. (2020) 533:533–9. doi: 10.1016/j.bbrc.2020.09.003 32988584

[28] Liu L Yue Q Ma J Liu Y Zhao T Guo W. POU4F1 promotes the resistance of melanoma to BRAF inhibitors through MEK/ERK pathway activation and MITF up-regulation. Cell Death Dis. (2020) 11:451. doi: 10.1038/s41419-020-2662-2 32532957 PMC7293281

[29] Yan Y Teng H Hang Q Kondiparthi L Lei G Horbath A. SLC7A11 expression level dictates differential responses to oxidative stress in cancer cells. Nat Commun. (2023) 14:3673. doi: 10.1038/s41467-023-39401-9 37339981 PMC10281978

