# Supplementary material for: Identifying and evaluating a disulfidptosis-related gene signature to predict prognosis in colorectal adenocarcinoma patients
Source: Front Immunol. 2024 Jun 19;15:1344637. doi: 10.3389/fimmu.2024.1344637 (PMC11220892; doi:10.3389/fimmu.2024.1344637)
Supplement: Supplementary file 1 [file DataSheet_1.pdf]

Identifying and Evaluating of a Disulfidptosis-Related Genes Signature to Predict Prognosis in Colorectal Adenocarcinoma Patients

Ming Li[1,2,3†], Jin Wang[4†], Yuhao Zhao[3†], Changjie Lin[1], Ke Tao[2], Pengcheng Zhu[2], Qi Hu[2], Jianfeng Gu[2*], Shaohua Wei[1*]

1 Department of General Surgery, The Second Affiliated Hospital of Soochow University, SanXiang Road 1055, Suzhou, 215000, China.

2 Department of General Surgery, Changshu Hospital Affiliated to Soochow University, The First People's Hospital of Changshu, Shuyuan Street 1, Changshu, 215500, Jiangsu, China.

3 Department of Biliary and Pancreatic Surgery, Renji Hospital Affiliated to Shanghai Jiaotong University School of Medicine, 160 Pujian Road, Pudong New District, Shanghai, 200127, China

4 School of Public Health, Suzhou Medical College of Soochow University, Suzhou, 215123, Jiangsu, China

† These authors contributed equally to this work as co-first authors.

Table S1. DRGs risk score was related to clinic-pathological features in GSE39582 dataset

Characteristics		Frequency	Mean ± SD	P value
Age	> 60	404	1.483 ± 1.135	0.197
	≤ 60	157	1.339 ± 1.205	
Gender	Female	253	1.426 ± 1.164	0.770
	Male	309	1.454 ± 1.148	
Location	Distal	342	1.421 ± 1.116	0.604
	Proximal	220	1.474 ± 1.214	
MMR	dMMR	72	0.962 ± 1.199	**< 0.001**
	pMMR	444	1.565 ± 1.142	
TP53_MUT	MU	190	1.491 ± 1.117	0.472
	WT	159	1.403 ± 1.138	
KRAS_MUT	MU	216	1.544 ± 1.145	0.079
	WT	325	1.366 ± 1.153	
BRAF_MUT	MU	49	1.418 ± 1.281	0.980
	WT	459	1.423 ± 1.158	
CIN_status	Negative	108	1.326 ± 1.247	0.515
	Positive	352	1.413 ± 1.091	
TNM_stage	Stage I	32	0.733 ± 1.298	**< 0.001**
	Stage II	262	1.317 ± 1.107	
	Stage III	204	1.482 ± 1.057	
	Stage IV	60	2.225 ± 1.231	
TNM_M	M0	479	1.328 ± 1.111	**< 0.001**
	M1	61	2.201 ± 1.235	
TNM_N	N0	299	1.271 ± 1.155	**0.004**
	N1	133	1.558 ± 1.132	
	N2	98	1.714 ± 1.138	
	N3	6	1.881 ± 0.760	
TNM_T	T0	1	1.145 ± NA	**< 0.001**
	T1	11	0.597 ± 1.434	
	T2	44	1.132 ± 1.201	
	T3	364	1.343 ± 1.084	
	T4	119	1.875 ± 1.214	

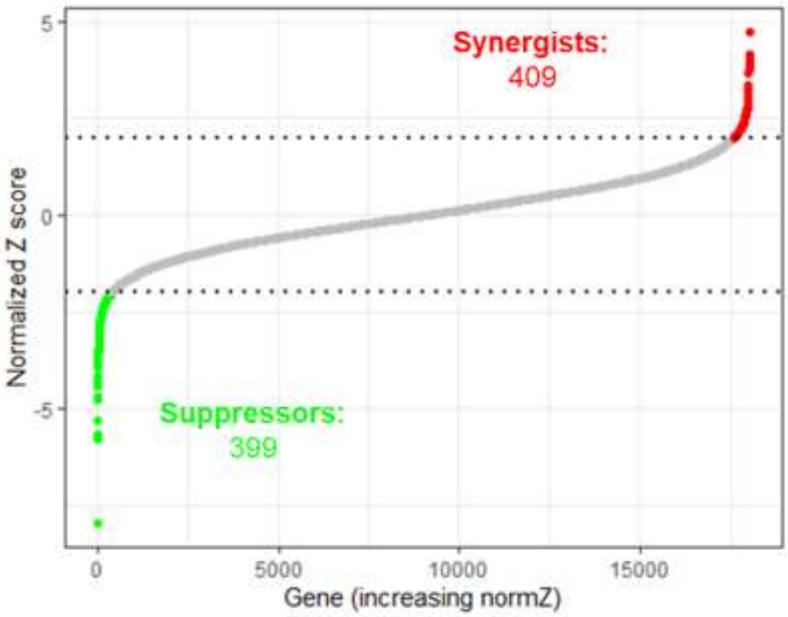

Figure S1. Scatter plot shows the DRGs screened based on the CRISPR–Cas9 screenings. DRGs: disulfidptosis-related genes.

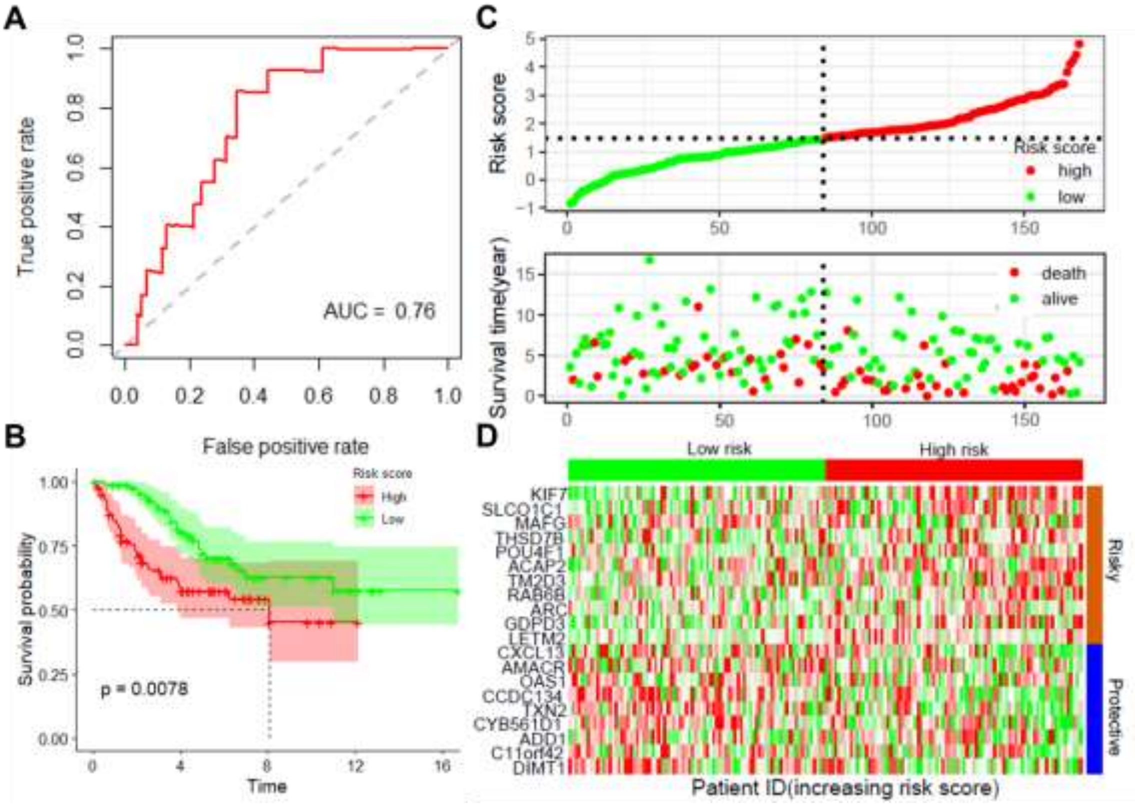

Figure S2. Validation of the prognostic model with 20 DRGs constructed by training dataset in internal testing dataset. (A) ROC curves for 5-year overall survival in internal testing dataset. (B) Kaplan–Meier curve of overall survival in internal testing dataset. (C) Risk score distribution and survival status of internal testing dataset. (D) Heatmap shows the expression of 20 DRGs in internal testing dataset. DRGs: disulfidptosis-related genes; ROC: receiver operating curve; COAD: Colon adenocarcinoma.

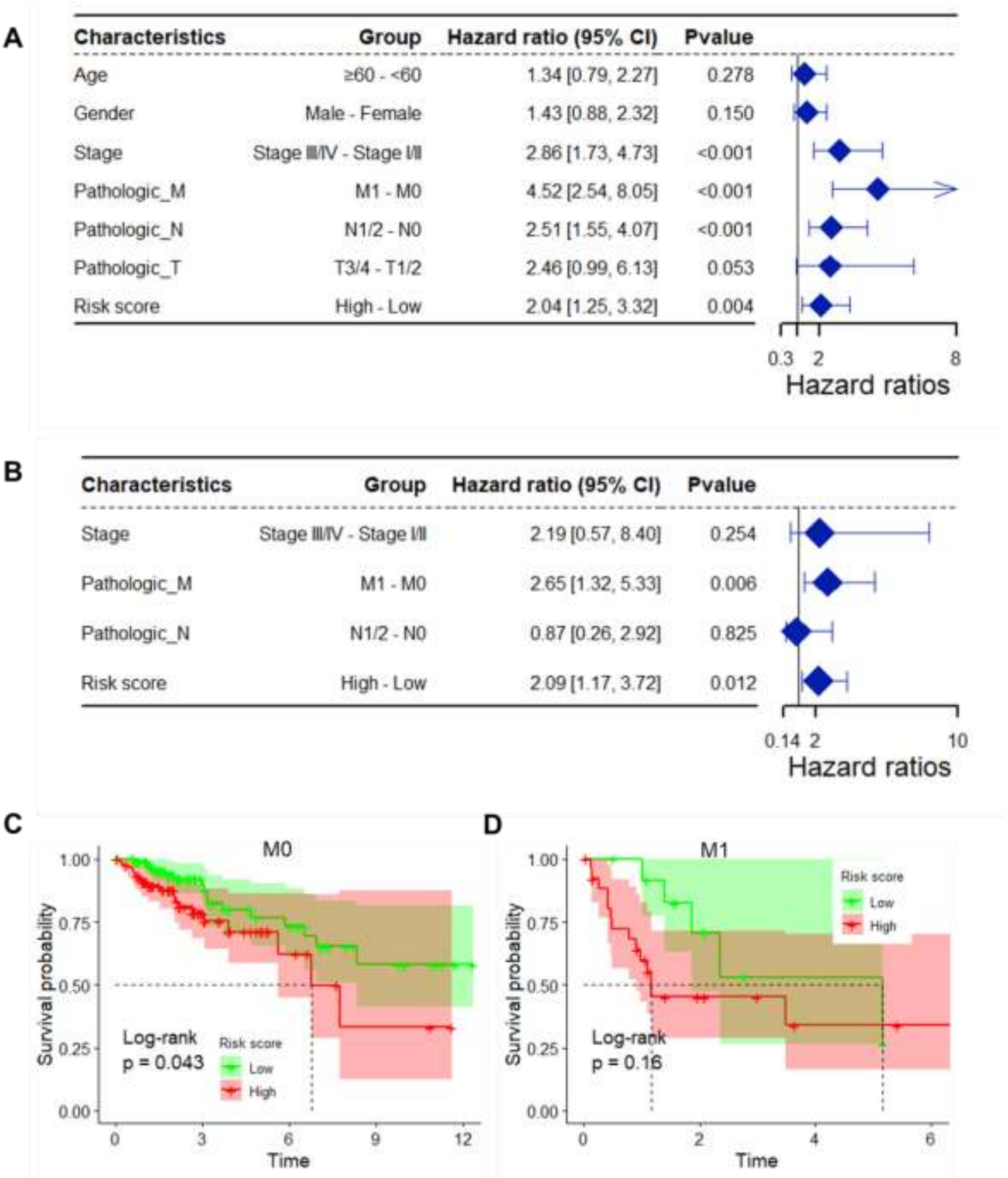

Figure S3. DRGs risk score is an independent prognostic factor for overall survival in the entire GSE39582 dataset. Univariate (A) and multivariate (B) Cox regression analyses of risk score and clinic-pathological features for overall survival in the entire GSE39582 dataset. (C-D) Kaplan-Meier analysis of overall survival stratified by TMN_M stage. DRGs: disulfidptosis-related genes.

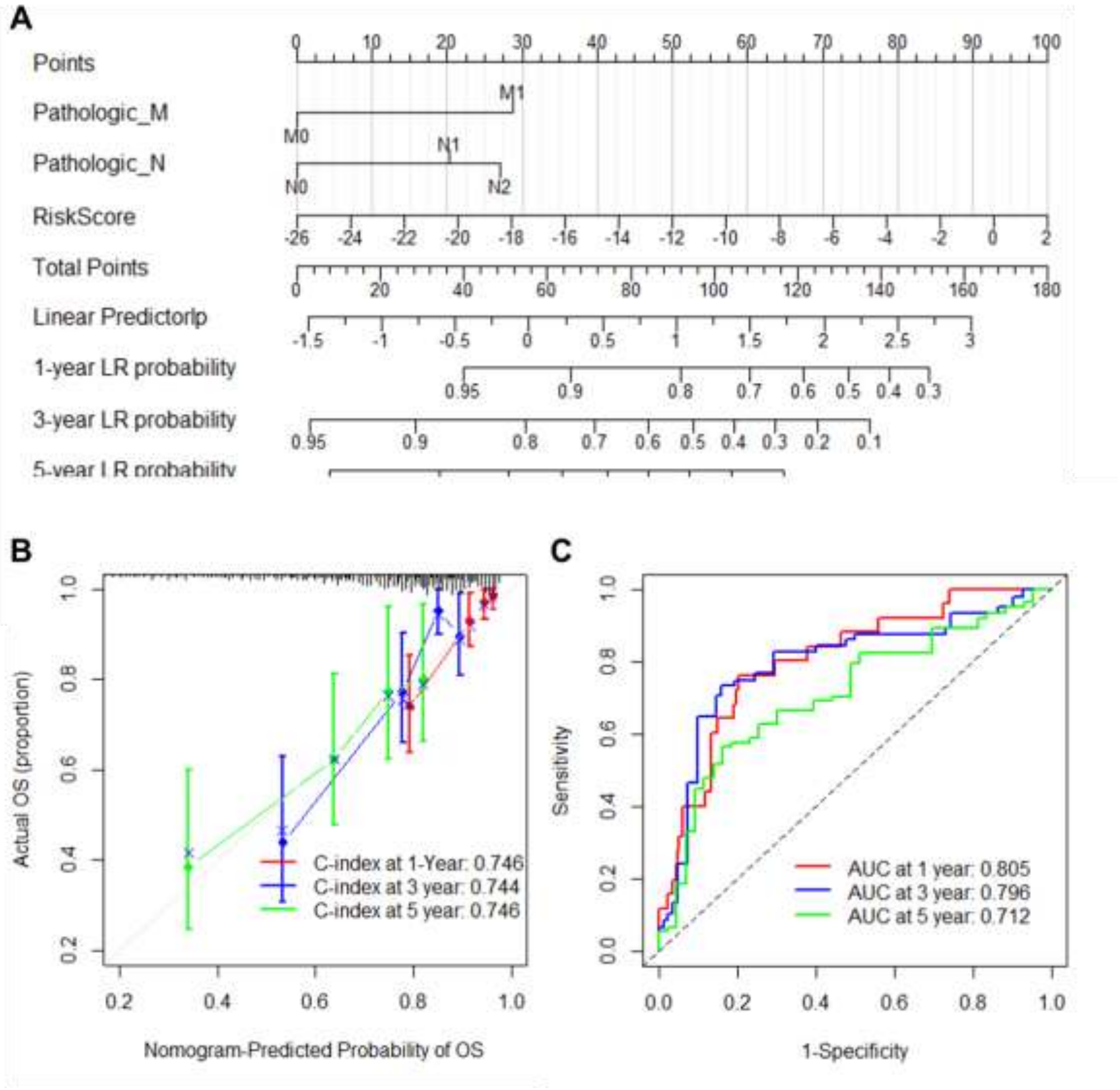

Figure S4. Nomogram predicting 1-year, 3-year and 5-year overall survival of COAD patients based on TCGA-COAD dataset. (A) The nomogram consists of the 20-DRGs risk score and 12 clinical indicators based on entire TCGA-COAD dataset. Add the points from these variables together and find the location of the Total Points. The Total Points projected on the bottom scales indicate the probability of 1-year, 3-year and 5-year overall survival. (B) 1/3/5-year calibration plot validating the accuracy of a prognostic nomogram based on the TCGA-COAD dataset. (C) ROC curves of overall survival for the score calculated from nomogram in TCGA-COAD dataset. DRGs: disulfidptosis-related genes. COAD: colon adenocarcinoma.

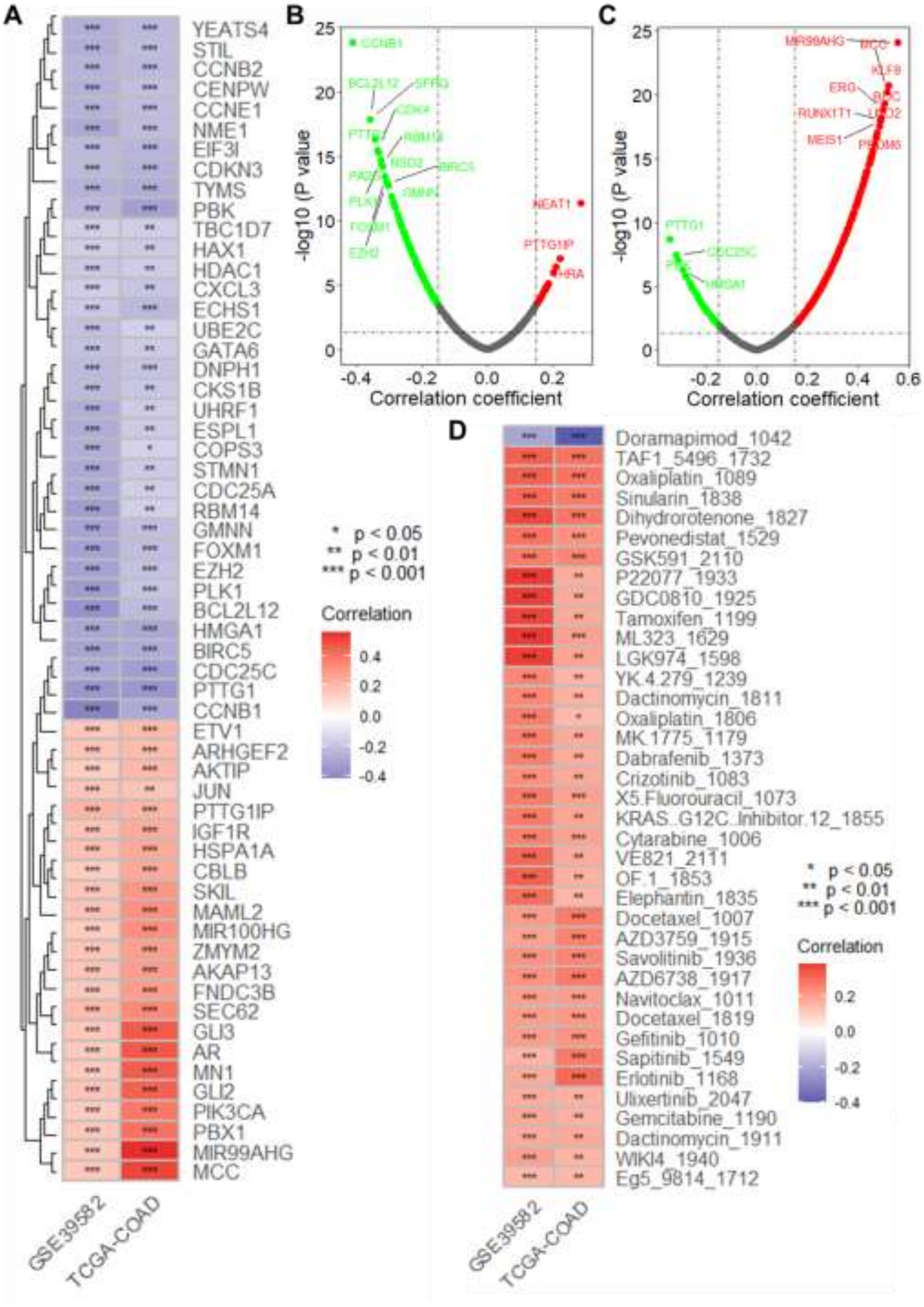

Figure S5. Risk score is significantly correlated with the expression of multiple oncogenes and the sensitivity of multiple anti-tumor drugs. (A) Heatmap shows the intersection of the oncogenes correlated with risk score in TCGA-COAD and GSE39582 datasets. Volcano plots show the correlation between DRGs risk score and the expression of oncogenes in GSE39582 (B) and TCGA-COAD (C) datasets. (D) The heatmap shows the intersected anti-tumor drugs associated with DRGs risk score in the TCGA-COAD and GSE39582 datasets. COAD: colon adenocarcinoma.

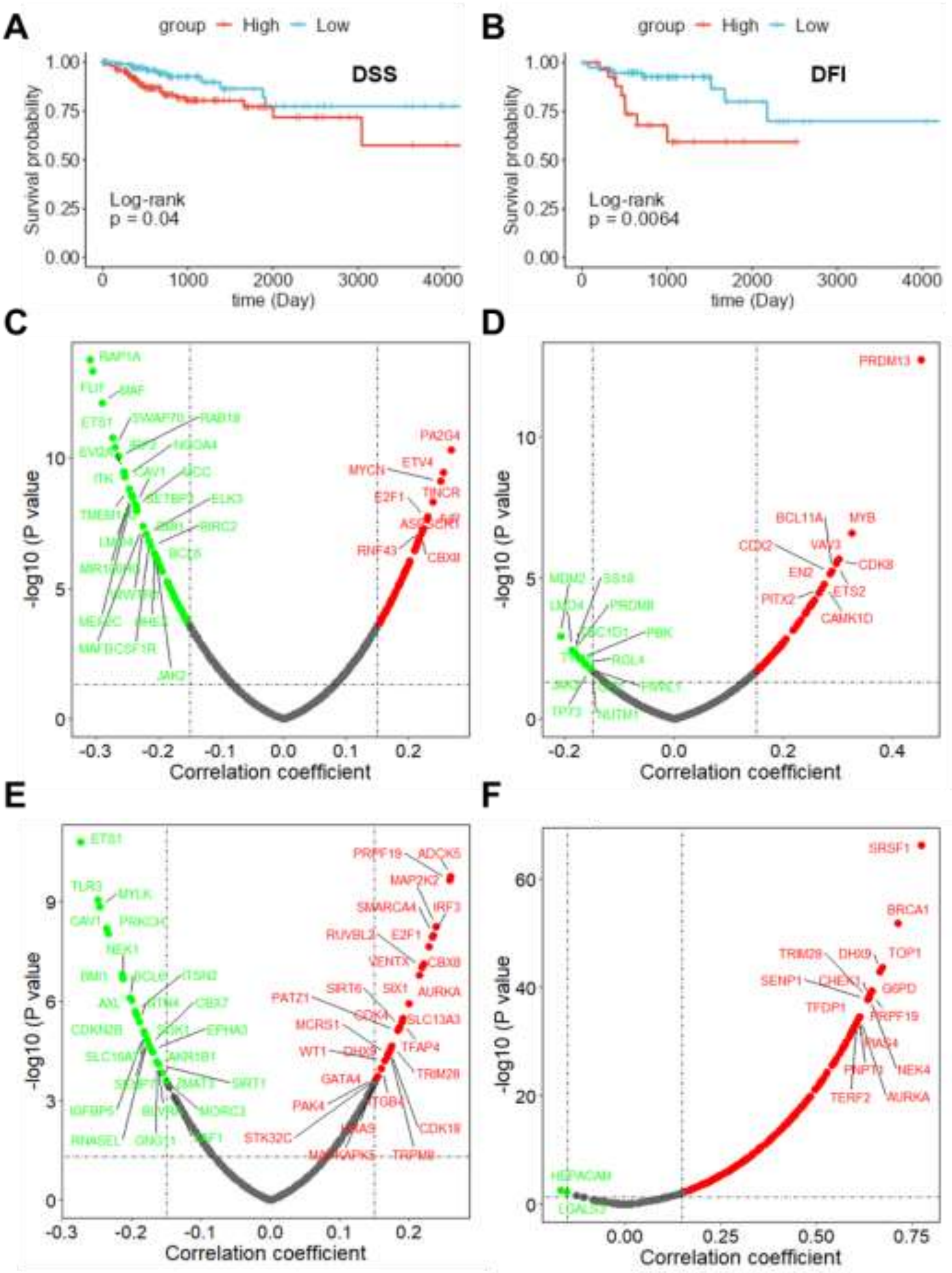

Figure S6. Kaplan-Meier analysis of disease-specific survival (A) and disease-free survival (B) stratified by POU4F1 expression, and the cutpoint is the median value. Volcano plots show the correlation between the expression of POU4F1 and oncogenes in GSE39582 (C) and TCGA-COAD (D) datasets. Volcano plots show the correlation between the expression of POU4F1 and cell senescence-related genes in GSE39582 (C) and TCGA-COAD (D) datasets. COAD: colon adenocarcinoma.

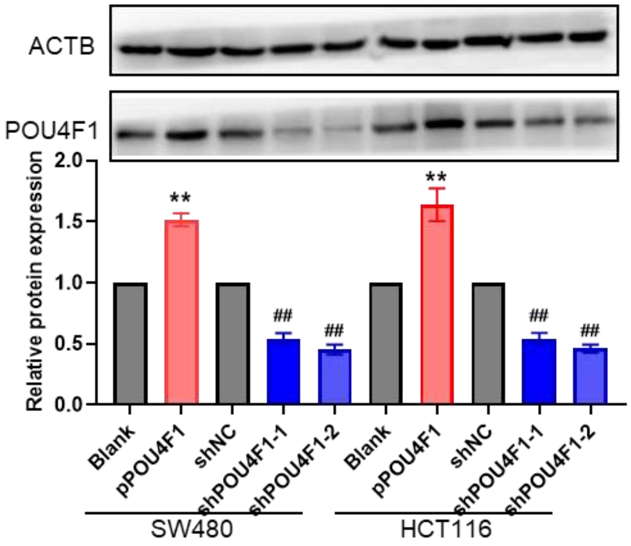

Figure S7. Western blotting was used to validate the protein expression in COAD cells transfected with POU4F1 overexpressed and knocked down plasmids.
